# New Inhibitory Effects of Cilnidipine on Microglial P2X7 Receptors and IL-1β Release: An Involvement in its Alleviating Effect on Neuropathic Pain

**DOI:** 10.3390/cells10020434

**Published:** 2021-02-18

**Authors:** Tomohiro Yamashita, Sawako Kamikaseda, Aya Tanaka, Hidetoshi Tozaki-Saitoh, Jose M. M. Caaveiro, Kazuhide Inoue, Makoto Tsuda

**Affiliations:** 1 Department of Global Healthcare, Graduate School of Pharmaceutical Sciences, Kyushu University, 3-1-1 Maidashi, Higashi-ku, Fukuoka 812-8582, Japan; jose@phar.kyushu-u.ac.jp; 2 Department of Molecular and System Pharmacology, Graduate School of Pharmaceutical Sciences, Kyushu University, 3-1-1 Maidashi, Higashi-ku, Fukuoka 812-8582, Japan; sawango1016@gmail.com (S.K.); tanakaaya.67n@gmail.com (A.T.); pdsaito@phar.kyushu-u.ac.jp (H.T.-S.); inoue@phar.kyushu-u.ac.jp (K.I.); 3 Department of Life Innovation, Graduate School of Pharmaceutical Sciences, Kyushu University, 3-1-1 Maidashi, Higashi-ku, Fukuoka 812-8582, Japan

**Keywords:** IL-1β, P2X7R, microglia, neuropathic pain, inflammation, cilnidipine, high-throughput screening

## Abstract

P2X7 receptors (P2X7Rs) belong to a family of ATP-gated non-selective cation channels. Microglia represent a major cell type expressing P2X7Rs. The activation of microglial P2X7Rs causes the release of pro-inflammatory cytokines such as interleukin-1β (IL-1β). This response has been implicated in neuroinflammatory states in the central nervous system and in various diseases, including neuropathic pain. Thus, P2X7R may represent a potential therapeutic target. In the present study, we screened a chemical library of clinically approved drugs (1979 compounds) by high-throughput screening and showed that the Ca^2+^ channel blocker cilnidipine has an inhibitory effect on rodent and human P2X7R. In primary cultured rat microglial cells, cilnidipine inhibited P2X7R-mediated Ca^2+^ responses and IL-1β release. Moreover, in a rat model of neuropathic pain, the intrathecal administration of cilnidipine produced a reversal of nerve injury-induced mechanical hypersensitivity, a cardinal symptom of neuropathic pain. These results point to a new inhibitory effect of cilnidipine on microglial P2X7R-mediated inflammatory responses and neuropathic pain, proposing its therapeutic potential.

## 1. Introduction

Neuropathic pain is a chronic pain condition caused by nerve injury or diseases such as diabetes, cancer, fibromyalgia, chemotherapy, infection, and autoimmune diseases. Neuropathic pain significantly impairs quality of life and is often resistant to known treatments, including opioids [1]. An increasing body of literature indicates that neuropathic pain is associated with the inflammatory milieu of the central nervous system (CNS) after nerve injury. Microglia, the resident immune cells of the CNS, are central players in neuroinflammation. Activated microglia are observed in the spinal cord and brain in diverse models of neuropathic pain, including peripheral nerve injury (PNI) [2,3,4], and are known to release various inflammatory mediators including proinflammatory cytokines, critically contributing to the development of neuroinflammatory milieu in the CNS, alongside behavioral pain hypersensitivity [5,6].

The ATP-gated P2X7 receptor (P2X7R), which belongs to the P2X receptor (P2XR) family, plays a key role in inflammation and immunity in the CNS through its action on microglia. Activation of microglial P2X7Rs causes the production and release of various inflammatory factors, including IL-1β and other proinflammatory cytokines and chemokines [7,8]. An efflux of intracellular K^+^ and influx of extracellular Ca^2+^ via P2X7Rs has been shown to potently activate NOD-leucine rich repeat and pyrin containing protein 3 (NLRP3) inflammasomes by combination of activation of Toll-like-receptor 4 (TLR4) [9]. Activated caspase-1 leads to the cleavage and release of IL-1β. In spinal cord microglia, P2X7R has been shown to be responsible for ATP-induced IL-1β release [10]. Consistently, mice lacking P2X7Rs exhibit reduced pain hypersensitivity after PNI [11], and the pharmacological blockade of P2X7Rs also produced a suppressive effect on pain hypersensitivity in rodent models of neuropathic pain [12,13,14]. PNI increases P2X7R expression in the spinal dorsal horn, and microglia are the predominant cell type [15,16]. Therefore, P2X7R is a promising target for treating neuropathic pain [17,18,19]. Despite recent progress in the development of P2X7R-selective antagonists, there are currently no approved drugs targeting P2X7R used in clinical practice and no proof of concept for the potential translation of P2X7R-targeted pain therapies.

One approach for facilitating translation and providing a proof of concept could be to use clinically approved drugs. Indeed, recent studies have pointed to new unexpected pharmacological effects of approved drugs and have provided potential insights into their eventual repurposing [20]. As for P2X7R, several drugs have been reported to have an inhibitory effect on P2X7R [21,22,23,24], but their effects require a high concentration. Therefore, in this study, we screened, for the first time, clinically approved drugs from a large-scale chemical library to identify those that have an inhibitory effect on P2X7R activation and identified cilnidipine, a Ca^2+^ channel blocker that inhibits P2X7R, microglial IL-1β release, and behavioral hypersensitivity in a rat model of neuropathic pain.

## 2. Materials and Methods

### 2.1. Reagents

Reagents were obtained from the following sources: adenosine 5′-triphosphate disodiumsalt (ATP; A7699), 2′(3′)-O-(4-Benzoylbenzoyl)adenosine 5′-triphosphate triethylammonium salt (BzATP; B6396), lipopolysaccharide (LPS; L4391), A-438079 hydrochloride hydrate (A-438079; A9736), Brilliant Blue G pure (BBG; B0770), benzbromarone (B5774), zafirlukast (Z4152) and cilnidipine (C1493) from Sigma-Aldlich, St. Louis, MO, USA; Celiprolol Hydrochloride (sc-211051) and mizolastine (sc-358366) from Santa Cruz Biotechnology, Inc., Paso Robles, CA, USA; Fluo 4-AM and Fura 2-AM from Dojindo, Kumamoto, Japan; YO-PRO-1 iodide 491/509 (YO-PRO-1; Y3603) and Pluronic F-127 (Pluronic; P3000MP) from Thermo Fisher Scientific Inc., Waltham, MA, USA; Poly-L-lysine hydrobromide MW 30,000–73,000 (Poly-L-lysine; 169-19071) and phorbol-12-myristate 13-acetate (PMA; 162-23591) from FUJIFILM Wako Pure Chemical Corporation (Wako), Osaka, Japan.

### 2.2. Cell Cultures

1321N1 human astrocytoma cells stably expressing rat P2X7R (rP2X7R-1321N1) and human P2X7R (hP2X7R-1321N1) were developed using a lentiviral CS2 vector [25] encoding rP2X7R (NCBI Reference Sequence: NM_019256) or hP2X7R (NCBI Reference Sequence: NP_002553 [Glu496Ala polymorphism]) driven by the human elongation factor 1α (EF1α) promoter (containing the puromycin-resistant gene). Cells were maintained in low-glucose Dulbecco’s modified Eagle’s medium (DMEM; Thermo Fisher Scientific, Inc.) supplemented with 10% heat-inactivated fetal bovine serum (FBS), penicillin, and streptomycin. Rat primary cultured microglia were prepared according to a previously reported method [26]. In brief, mixed glial cultures were prepared from the brain of neonatal Wistar rats (Kyudo Japan) and maintained for 9–24 days in high-glucose DMEM (Thermo Fisher Scientific, Inc.) supplemented with 4 mM GlutaMax, 10% heat-inactivated FBS, penicillin and streptomycin. THP-1 cells (human monocytic cells) were maintained in RPMI 1640 medium (Wako) supplemented with 10% heat-inactivated FBS, penicillin, and streptomycin. Before using the ELISA assay, THP-1 cells were differentiated by a 3-h incubation with 0.5 μM PMA.

### 2.3. Animals

Male Wistar adult rats (250–280 g; Japan SLC) were housed in individual cages. Neonatal Wistar rats (Kyudo, Japan) were used for the experiments in primary cultured microglia. The animals were housed in groups of four per cage with wooden chips on the floor during habituation, after which they were placed in individual cages after intrathecal catheterization under controlled temperature (22 ± 1 °C) and humidity (55 ± 10%). The room was lit up from 8:00 a.m. to 8:00 p.m. All animals were provided food and water ad libitum. All animal experiments were conducted according to relevant national and international guidelines contained in the ‘Act on Welfare and Management of Animals’ (Ministry of Environment of Japan) and ‘Regulation of Laboratory Animals’ (Kyushu University) and according to the protocols approved by the Institutional Animal Care and Use committee review panels at Kyushu University.

### 2.4. Evaluation of rP2X7R Inhibition by High-Throughput Screening

A library composed of 1979 compounds was provided in 96-well plate format by the Drug Discovery Initiative (DDI) of the University of Tokyo, which possesses the largest collection of compound library in Japan. Evaluation of rP2X7R inhibition was performed in the Functional Drug Screening System 7000EX (FDSS7000EX; Hamamatsu, Japan) by Ca^2+^ imaging. The rP2X7R-1321N1 cells were cultured for 24 h at 37 °C in 96-well plates (2.0 × 10^4^ cells/well). Cells were loaded with 2.5 μM Fluo 4-AM containing 0.04% pluronic in balanced salt solution (BSS; 150 mM NaCl, 5 mM KCl, 1.8 mM CaCl_2_, 1.2 mM MgCl_2_, 10 mM D-glucose, and 25 mM HEPES, pH7.4) at room temperature for 45 min and then washed with BSS. After pretreatment with 10 μM compounds for 10 min, cells were stimulated with BzATP (prepared at 5-fold concentration) at a final concentration of 60 μM. Data plots data were normalized with respect to a control sample (60 μM BzATP induced Ca^2+^ responses). Evaluation of the chemical library was carried out in only one trial. All assays qualified the criteria for the screening of large compound libraries (coefficient of variation (CV) < 10%, Z’ values > 0.5).

### 2.5. Measurement of Ca^2+^ Responses

Ca^2+^ imaging apparatus in 96-well plates were performed using the FDSS7000EX. The rP2X7R-1321N1 or hP2X7R-1321N1 were cultured for 24 h at 37 °C in 96-well plates (2.0 × 10^4^ cells/well). Cells were loaded with 2.5 μM Fluo 4-AM containing 0.04% pluronic in BSS at room temperature for 45 min and then washed with BSS. After pretreatment with each compound for 10 min, cells were stimulated with BzATP (prepared at 5-fold concentration) at a final concentration of 60 or 100 μM. Ca^2+^ responses in single cells were assessed by ratiometric images (F340/F380) of Fura 2 fluorescence, which were detected with Aquacosmos/HiSca (Hamamatsu Photonics, Hamamatsu, Japan). The rP2X7R-1321N1 or rat primary microglial cells were cultured for 24 or 3 h at 37 °C on 10 µg/mL poly-L-lysine-coated Flexiperm cover glass (1.0 or 5.0 × 10^4^ cells/well, respectively). Cells were loaded with Fura 2-AM in BSS or DMEM at 37 °C for 30 min. Cells were then washed with BSS and mounted on an inverted fluorescence microscope (ECLIPSE TE2000-U; Nikon) equipped with a xenon lamp (Xe75W; Nikon). The inhibitory effects of compounds in rP2X7R-1321N1 were evaluated using the S2 (2nd [Ca^2+^] response)/S1 (1st [Ca^2+^] response) ratio. After pretreatment with 0.3–10 μM compounds for 10 min, cells were stimulated with BzATP (100 μM for 20 s). After washing out the compounds with BSS, we confirmed the recovery of Ca^2+^ responses by a third BzATP stimulation. The inhibitory effects of 10 μM compounds in rat primary microglia were evaluated compared to only ATP (2 mM for 30 s) or BzATP (100 μM for 30 s) induced Ca^2+^ responses.

### 2.6. YO-PRO-1 Dye Uptake Assay 

The rP2X7R-1321N1 or hP2X7R-1321N1 were cultured for 24 h at 37 °C in 96-well plates (2.0 × 10^4^ cells/well). Cells were incubated with 2 μM YO-PRO-1 and 10 μM compounds in Hanks’ Balanced Salt Solution, without any calcium or magnesium (HBSS (–); Thermo Fisher Scientific, Inc.), with 0.1 mM CaCl_2_ at room temperature for 10 min. After incubation, BzATP was added at a final concentration of 60 μM. Fluorescence intensity was measured using a plate reader (EnSpire; Perkin Elmer Japan) for 30 min. The basal line and measurements were recorded with an excitation wavelength of 491 nm and an emission wavelength of 509 nm.

### 2.7. Western Blotting Analysis

To detect precursor and mature IL-1β expression, LPS (500 ng/mL for 3 h)-primed rat microglial cells were treated with compounds (10 μM for 30 min) and then treated with ATP (2 mM for 30 min, co-treatment with each compound). After stimulation, cells (lysates) and culture medium (supernatants) were isolated, and each sample lysed in RIPA buffer (Nacalai Tesque, Inc., Kyoto, Japan). The samples were subjected to a 10% polyacrylamide gel, and the proteins were transferred electrophoretically to PVDF membranes (GE Healthcare, Tokyo, Japan). After blocking with blocking buffer (Nacalai Tesque, Inc., Kyoto, Japan), membranes were incubated with anti-IL-1β, rat, goat-poly (1:1000; AF-501-NA, R&D systems) and β-actin (1:5000; Sigma-Aldlich, St. Louis, MO, USA). The antibodies were detected using an HRP-conjugated secondary antibody (1:1000, GE Healthcare, Tokyo, Japan) and visualized using an ECL system (GE Healthcare, Tokyo, Japan).

### 2.8. ELISA Assay (Measurement of Interleukin-1β (IL-1β) Release)

Rat primary cultured microglia (2 × 10^5^ cells/well) were cultured in 24-well plates for 1 h, after which the cells were incubated for 1 h with fresh serum-free medium. LPS (500 ng/mL for 3 h)-primed microglial cells were treated with compounds (1–10 μM for 30 min) and then stimulated with ATP (2 mM for 30 min, co-treated with each compound). PMA-differentiated THP-1 cells (2 × 10^5^ cells/well) were cultured in 24-well plates for 24 h. LPS (500 ng/mL for 3 h)-primed cells were treated with compounds (10 μM for 30 min) and then stimulated with BzATP (300 μM for 30 min, co-treatment with each compound). The supernatants were used to measure the IL-1β release. The concentration of IL-1β in each sample was determined using the Rat IL-1 beta/IL-1F2 Quantikine ELISA Kit (RLB00, R&D Systems, Minneapolis, MN, USA) or Human IL-1 beta/IL-1F2 Quantikine ELISA Kit (DLB50, R&D Systems, Minneapolis, MN, USA). Each assay was performed in accordance with the manufacturer’s instructions.

### 2.9. Intrathecal Administration

Under isoflurane (2% [*v*/*v*]) anesthesia, a 32-gauge intrathecal catheter (ReCathCo, Allison Park, PA, USA) for intrathecal administration of cilnidipine was inserted through the atlanto-occipital membrane into the lumbar enlargement and externalized through the skin according to a previously described method [27,28].

### 2.10. Neuropathic Pain Model and Behavioral Test

The spinal nerve injury model was prepared according to a previously reported method [29]. Experiments were performed using adult male Wistar rats (250–280 g; Japan SLC). A unilateral L5 spinal nerve was tightly ligated and cut just distal to the ligature under isoflurane (2.5%) anesthesia. To assess mechanical pain hypersensitivity, calibrated von Frey filaments (0.4–15 g, Linton Instrumentation) were applied to the plantar surface of the hind paw from below the mesh floor. The 50% paw-withdrawal threshold (PWT) was determined using the up-down method. Cilnidipine (100 ng/10 μL of phosphate-buffered saline) or vehicle (phosphate-buffered saline, 10 μL) was intrathecally administered to rats 7 days post-PNI or once a day for 7 days from 1 day post-PNI; mechanical pain hypersensitivity was measured for 180 min.

### 2.11. Statistical Analysis

Statistical analyses were performed using a Student’s t-test (an ELISA assay and behavioral tests), a one-way ANOVA with Dunnett’s or a Bonferroni’s multiple comparison test (measurements of Ca^2+^ responses, YO-PRO-1 dye uptake assay, and an ELISA assay) or a two-way ANOVA with a post hoc Bonferroni test (behavioral tests) using GraphPad Prism 4 software. Differences were considered significant for values of *p* < 0.05.

## 3. Results

### 3.1. Identification of Several Compounds That Inhibit P2X7R Function by High-Throughput Screening

**Figure 1:**
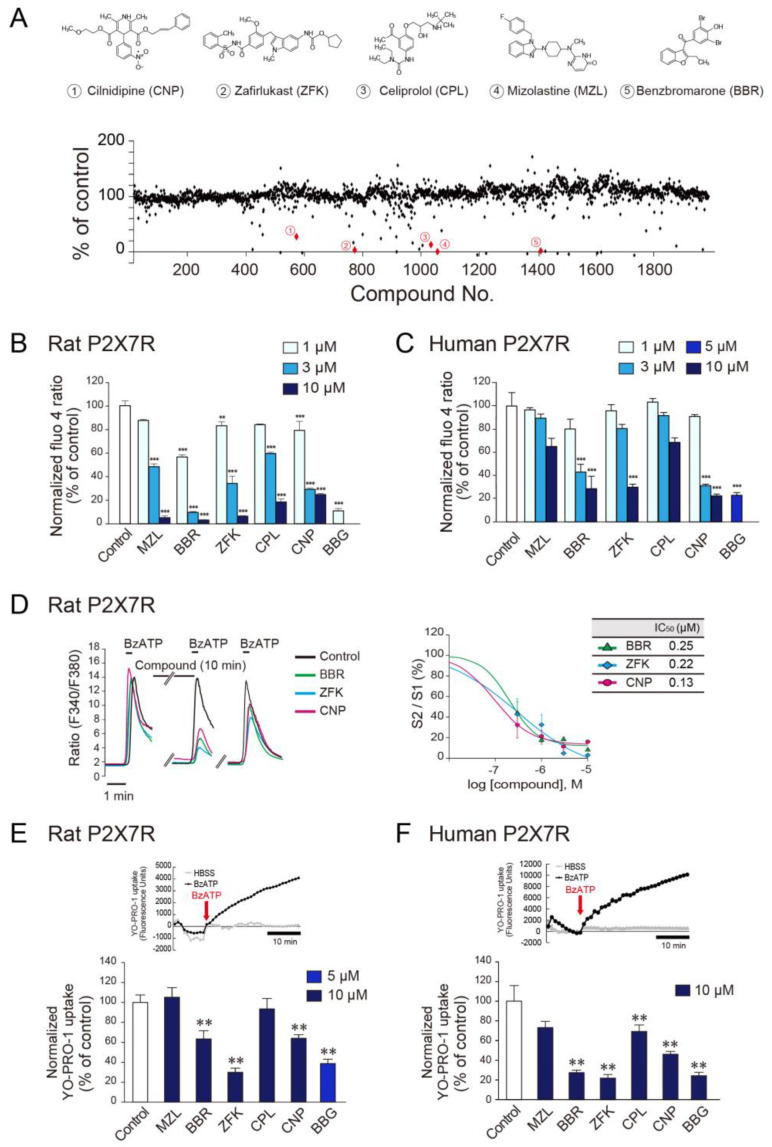
Compounds identified by high-throughput screening inhibit the function of recombinant P2X7R in 1321N1 cells. (**A**) Scatter plot of the % inhibitory efficacy of 1979 compounds. Highlighted in red are compounds validated to strongly inhibit BzATP (60 μM)-induced Ca^2+^ responses in rP2X7R-1321N1 cells. Chemical structures of the five compounds focused on in this study. (**B**) Inhibitory effects of each compound (1–10 μM; mizolastine (MZL), benzbromarone (BBR), zafirlukast (ZFK), celiprolol (CPL), cilnidipine (CNP)) or 1 μM BBG on BzATP (60 μM)-induced Ca^2+^ responses in rP2X7R-1321N1 cells (*n* = 4–6, ** *p* < 0.01, *** *p* < 0.001 vs. control). (**C**) Inhibitory effects of each compound (1–10 μM) or 5 μM BBG on BzATP (100 μM)-induced Ca^2+^ responses in hP2X7R-1321N1 cells (*n* = 3–6, *** *p* < 0.001 vs. control). (**D**) Effect of compounds at various concentrations on BzATP (100 μM for 20 s)-induced Ca^2+^ responses in rP2X7R-1321N1 cells measured at a single cell level (*n* = 3–4). (**E**) The representative traces of YO-PRO-1 uptake evoked by BzATP (60 μM) in rP2X7R-1321N1 cells. Inhibitory effects of each compound (10 μM) or 5 μM BBG on YO-PRO-1 uptake stimulated by BzATP (60 μM) in rP2X7R -1321N1 cells (*n* = 4–9, ** *p*< 0.01 vs. control). (**F**) The representative traces of YO-PRO-1 uptake evoked by BzATP (60 μM) in hP2X7R-1321N1 cells. Inhibitory effects of each compound (10 μM) or 10 μM BBG on YO-PRO-1 uptake stimulated by BzATP (60 μM) in hP2X7R-1321N1 cells (*n* = 4–5, ** *p*< 0.01 vs. control). Data are represented as mean ± SEM.

To assess the effect of compounds on responses mediated by rat P2X7Rs, we established a cell line, 1321N1 cells, that stably expressed rat P2X7R (rP2X7R-1321N1) and measured intracellular Ca^2+^ increases induced by the P2X7R agonist BzATP using a high-throughput Ca^2+^ imaging apparatus. An increase in Ca^2+^ responses after application of BzATP was confirmed in rP2X7R-1321N1 cells [30], but not in native 1321N1 cells. We then evaluated 1979 compounds (10 μM) obtained from an approved drug library. Nine compounds suppressed the BzATP-induced Ca^2+^ responses by over 70% in rP2X7R-1321N1 cells and did not affect basal Ca^2+^ levels (Figure 1A). Among these compounds, five orally bioavailable drugs [mizolastine (a histamine receptor H1 antagonist), benzbromarone (a uric acid transporter inhibitor), zafirlukast (a leukotriene receptor antagonist), celiprolol (a β1-adrenoceptor antagonist), and cilnidipine (an L/N-type Ca^2+^ channel blocker)] and the P2X7R antagonist brilliant blue G (BBG; as a positive control) were selected, and their concentration-dependent inhibitory effects were confirmed (Figure 1A,B). In human P2X7R-1321N1 (hP2X7R-1321N1) cells, benzbromarone, zafirlukast, and cilnidipine also inhibited BzATP-induced Ca^2+^ responses, an effect that was similar to that of rP2X7R-1321N1 cells (Figure 1C). The inhibitory effect of these compounds was verified by single cell-based Ca^2+^ imaging, and IC50 values for benzbromarone, zafirlukast, and cilnidipine in rat P2X7R were 0.25 μM, 0.22 μM, and 0.13 μM, respectively (Figure 1D). We note that the IC50 values were lower than the concentrations at which inhibition was observed in Figure 1B. The likely reason was that we employed different approaches to determine their inhibitory effect. In addition, the single-cell data were obtained with a Ca^2+^ imaging apparatus that stimulates rat P2X7R expressed in 1321N1 cells by perfusing BzATP, whereas in Figure 1B we employed a single application of the agonist. P2X7R activation leads to the opening of a large pore formation, which is implicated in the release of IL-1β [10,31]. To examine the effect of the selected compounds on this change, we assessed the uptake of YO-PRO-1 in P2X7R-stimulated 1321N1 cells. We found that benzbromarone, zafirlukast, and cilnidipine significantly suppressed YO-PRO-1 uptake in both rP2X7R- and hP2X7R-1321N1 cells (Figure 1E,F). These data suggest that these clinically approved drugs have potent inhibitory effects on both rat and human P2X7Rs.

### 3.2. Benzbromarone, Zafirlukast, and Cilnidipine Inhibit the Responses of Rat Microglial Cells

**Figure 2:**
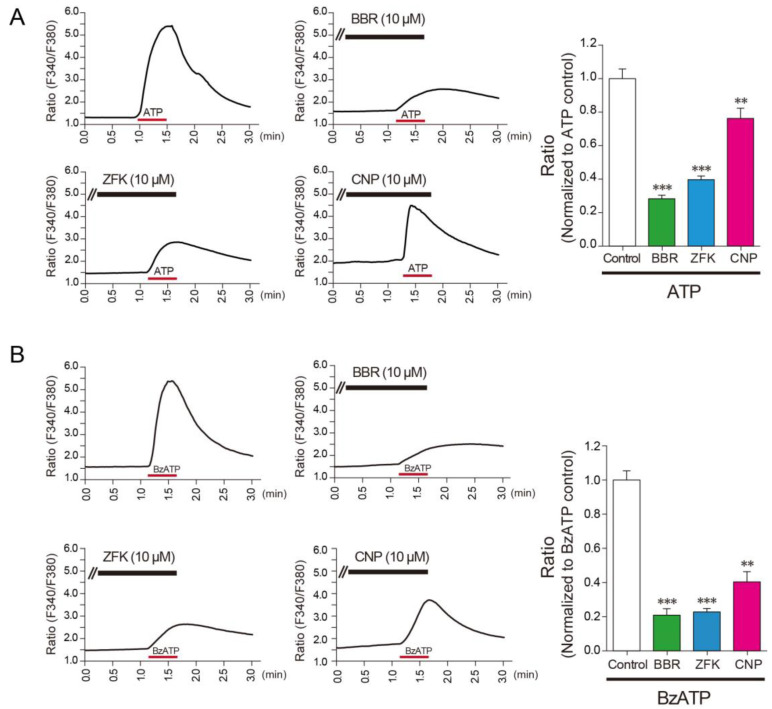
Benzbromarone, zafirlukast, and cilnidipine reduce ATP and BzATP-induced Ca^2+^ responses in rat microglial cells. (**A**) The representative average traces of ATP (2 mM for 30 s)-induced in response to with or without each compound (10 μM for 10 min; benzbromarone (BBR), zafirlukast (ZFK), cilnidipine (CNP)) measured by a single cell analysis in rat microglial cells. The inhibitory effect of each compound compared to ATP-induced Ca^2+^ responses in rat microglial cells (*n* = 4–6, ** *p* < 0.01, *** *p* < 0.001 vs. control). (**B**) The representative average traces of BzATP (100 μM for 30 s)-induced in response to with or without each compound (10 μM for 10 min; BBR, ZFK, CNP) measured by a single cell analysis in rat microglial cells. The inhibitory effect of each compound compared to BzATP-induced Ca^2+^ responses in rat microglial cells (*n* = 3–6, ** *p* < 0.01, *** *p* < 0.001 vs. control). Data are represented as mean ± SEM.

Based on our findings on recombinant P2X7R, we next examined the inhibitory effect of these compounds on rat primary cultured microglial cells that endogenously express P2X7Rs. Benzbromarone, zafirlukast, and cilnidipine significantly inhibited Ca^2+^ responses evoked by ATP and BzATP (Figure 2A,B), indicating that these compounds inhibit P2X7Rs endogenously expressed in microglia.

**Figure 3:**
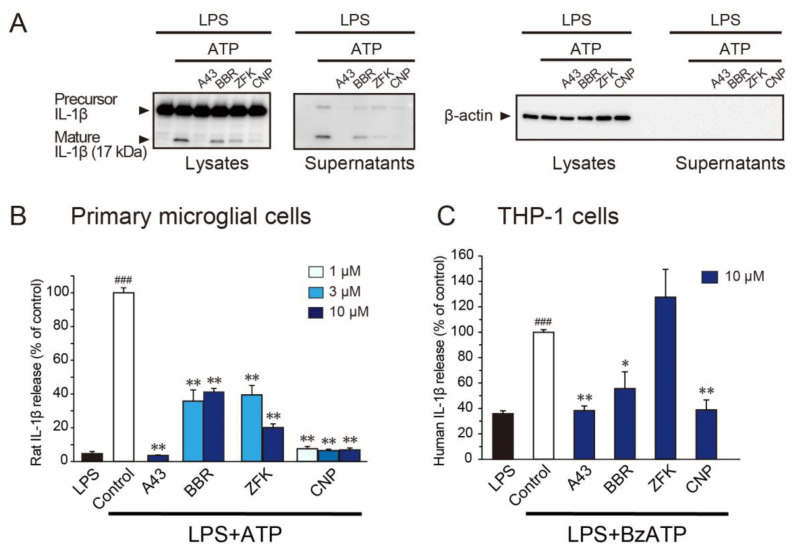
Benzbromarone, zafirlukast, and cilnidipine inhibit the release of mature IL-1β in rat microglial or THP-1 cells. (**A**) Western blot analysis of precursor and mature IL-1β in lysates (production) and supernatants (release) stimulated by LPS (500 ng/μL; 3 h) and ATP (2 mM; 30 min) in rat microglial cells. Each compound (10 μM; benzbromarone (BBR), zafirlukast (ZFK), cilnidipine (CNP), or A-438079 (A43; a selective P2X7R antagonist)) was treated 30 min before ATP stimulation. (**B**) The inhibitory effect of each compound compared to LPS (500 ng/μL; 3 h) and ATP (2 mM; 30 min)-induced release of mature IL-1β from rat microglial cells by ELISA analysis (*n* = 5–17, ^###^ *p* < 0.001 vs. LPS, ** *p* < 0.01 vs. control). (**C**) The inhibitory effect of each compound compared to LPS (500 ng/μL; 3 h) and BzATP (300 μM; 30 min)-induced release of mature IL-1β from PMA-differented THP-1 cells by ELISA analysis (*n* = 4–10, ^###^ *p* < 0.001 vs. LPS, * *p* < 0.05, ** *p* < 0.01 vs. control). Data are represented as mean ± SEM.

We next determined whether these three compounds affect the release of IL-1β from ATP-activated microglia that had been primed by lipopolysaccharide (LPS), a TLR4 agonist that is widely used to activate microglia and stimulate IL-1β release from spinal cord slices [32]. Level of mature IL-1β were induced in lysates and supernatants of primary cultured microglia stimulated by LPS (500 ng/μL; 3 h) and ATP (2 mM; 30 min) (Figure 3A), pointing to the production and release of IL-1β. Pretreatment with either benzbromarone, zafirlukast, or cilnidipine suppressed the levels of mature IL-1β in lysates and supernatants from LPS/ATP-stimulated microglia (Figure 3A). The suppressive effect of all compounds on mature IL-1β release was also confirmed by an ELISA assay (Figure 3B). Cilnidipine exerted the most potent inhibitory effect. Furthermore, cilnidipine significantly suppressed the release of mature IL-1β by BzATP stimulation in PMA-differentiated THP-1 cells (a human monocyte cell line) (Figure 3C). These data suggest that cilnidipine potently inhibits the release of IL-1β from microglial cells.

### 3.3. Cilnidipine Alleviates Mechanical Pain Hypersensitivity in a Model of Neuropathic Pain

**Figure 4:**
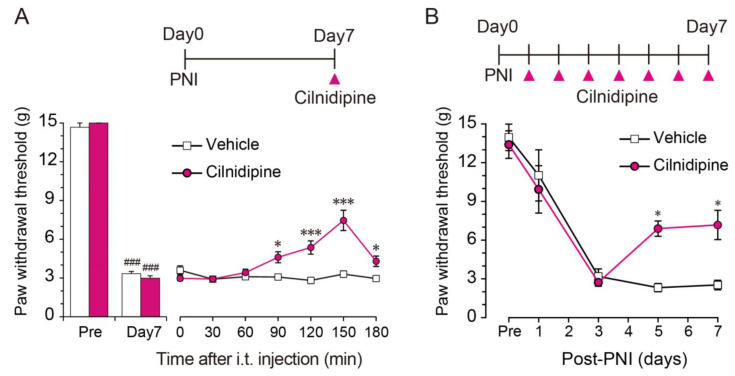
Intrathecal administered cilnidipine alleviates mechanical pain hypersensitivity after nerve injury. (**A**) Cilnidipine (100 ng/10 μL) or vehicle (phosphate-buffered saline, 10 μL) was intrathecally administered to rats on day 7 post-PNI (*n* = 9, ^###^ *p* < 0.001 vs. pre, * *p* < 0.05, *** *p* < 0.001 vs. vehicle). (**B**) A daily intrathecal of cilnidipine (100 ng/10 μL) or vehicle (phosphate-buffered saline, 10 μL) was administered from day 1 to day 7 to rats after nerve injury. The paw withdrawal threshold was measured at 150 min after intrathecal administered cilnidipine (*n* = 7–9, * *p* < 0.05 vs. vehicle). Data are represented as mean ± SEM.

Microglial P2X7Rs and IL-1β in the spinal cord contribute to neuropathic pain. Therefore, we selected cilnidipine to test its in vivo effect on neuropathic pain using a rat model in which the spinal nerve was ligated and transected (one of the most frequently used neuropathic pain models) [6,33]. Nerve injury resulted in a decrease in the threshold to withdraw the hindpaw from light mechanical stimulation on day 7, pointing to the development of mechanical pain hypersensitivity. Nerve injury-induced hypersensitivity was significantly suppressed by intrathecally administered cilnidipine (100 ng/10 μL) (Figure 4A). To test the effect on pain development, we administered a daily intrathecal of cilnidipine (100 ng/10 μL) in nerve injured rats from day 1 to 7 after nerve injury. The decrease in paw withdrawal threshold was not indistinguishable between vehicle- and cilnidipine-treated groups until day 3, but was significantly suppressed by cilnidipine treatment on days 5 and 7 (Figure 4B). These results suggest that cilnidipine has an alleviating effect on behavioral hypersensitivity associated with neuropathic pain.

## 4. Discussion

Drugs targeting P2X7R are currently being developed. Several P2X7R antagonists have been reported to date [34], but no drug has been approved clinically [18,35]. In this study, we screened a large number of clinically approved small chemicals for P2X7R by high-throughput Ca^2+^ imaging. We identified several compounds that have potent inhibitory effects on P2X7R. Among these chemicals, benzbromarone, zafirlukast, and cilnidipine were also effective against human P2X7R. Furthermore, their inhibitory effects on P2X7R-mediated responses were obtained in microglial cells from rats, suggesting that these compounds would inhibit microglial P2X7R in vivo. Moreover, previous studies reported some approved drugs that have an inhibitory effect on P2X7Rs [21,22,23,24]; however, the IC50 values of benzbromarone, zafirlukast, and cilnidipine for P2X7R were much lower (although these values were calculated from data obtained with a Ca^2+^ imaging apparatus that stimulates rat P2X7Rs expressed in 1321N1 cells by perfusing BzATP). We also found that mizolastine strongly inhibited Ca^2+^ response via rat P2X7R but not human P2X7R. It was reported that some P2X7R antagonists have different sensitivities between humans and rodent [34,36], suggesting that compounds to inhibit rat P2X7R do not necessarily have inhibitory effects for human P2X7R. Interestingly, among 22 blockers of voltage-dependent Ca^2+^ channels including the chemical library we tested, cilnidipine was the only compound that inhibited the BzATP-induced Ca^2+^ responses in rat P2X7R-expressing 1321N1 cells (Supplementary Figure S1), suggesting that the inhibitory effect of cilnidipine on P2X7R-mediated responses seems to be unique. Thus, our study is the first to screen small chemicals approved clinically and to identify several compounds that have potent inhibitory effects on P2X7R-mediated responses.

A key function of microglial P2X7R is the release of the proinflammatory cytokine IL-1β. Our study demonstrated that benzbromarone, zafirlukast, and cilnidipine effectively suppressed microglial IL-1β release induced by ATP. An unanticipated finding was that cilnidipine had the most potent inhibitory effect because its inhibition of P2X7R-mediated Ca^2+^ responses was slightly weaker than that of the other two compounds. It is thus conceivable that, in addition to P2X7R inhibition, other mechanisms could be involved in the effect of cilnidipine on microglial IL-1β release. One candidate for the other mechanism could be related to its action on dynamin-related protein 1 (DRP1), a mitochondrial fission factor that controls mitochondrial dynamics [37]. An imbalance in mitochondrial dynamics has been implicated in the activation of the NLRP3 inflammasome. In fact, it has been reported that DRP1-mediated mitochondrial fission leads to the activation of the NLRP3 inflammasome upon viral infection [38], and NLRP3 inflammasome activation is augmented by DRP1 knockdown [39]. Future work will determine whether cilnidipine affects inflammasome activation to act on DRP1.

The in vivo effect of cilnidipine was demonstrated in a model of neuropathic pain. Our data showed that intrathecal administration of cilnidipine attenuated mechanical pain hypersensitivity caused by nerve injury. This was supported by previous observations that microglia become activated after nerve injury and that P2X7R is required for ATP-induced IL-1β release from activated spinal microglia [32]. It has also been shown that the expression of P2X7R is selectively increased in spinal microglia on day 5 (but not on day 3) after nerve injury [15]. This time-course of microglial P2X7R upregulation in the spinal cord seems to be similar to that of the suppressive effect on mechanical pain hypersensitivity by intrathecal cilnidipine, an effect that was observed on day 5, but not on day 3. Considering that IL-1β facilitates excitatory synaptic transmission in the spinal dorsal horn [40], the in vivo effect of cilnidipine in this model of neuropathic pain may be linked to its inhibitory action on microglial P2X7Rs and IL-1β release. However, it seems unlikely that the inhibition of microglial P2X7Rs and IL-1β release is the sole mechanism by which cilnidipine suppresses neuropathic pain. Indeed, cilnidipine blocks L/N-type Ca^2+^ channels, which that have been shown to be expressed in the spinal terminals of primary afferent nociceptors and are implicated in nociceptive synaptic transmission [41]. Intrathecal injection of cilnidipine has been reported to rapidly produce an analgesic effect on nociceptive behavior and mechanical pain hypersensitivity in models of peripheral tissue injury [42] and nerve injury [43]. Although the N-type Ca^2+^ channel blocker conotoxin ω-conotoxin GVIA, but not the L-type Ca^2+^ channel blocker nicardipine, also attenuates these pain behaviors [42,43], interestingly, these reports also showed that the time-course of cilnidipine’s analgesic effects is distinct from that of ω-conotoxin GVIA. This suggests a mechanism other than a Ca^2+^ channel blockade. Therefore, it is conceivable that cilnidipine has a new analgesic mechanism through microglial P2X7R inhibition. 

Previous studies have reported a decrease in blood pressure by oral administration of cilnidipine (30 mg/kg) in rats [44], a dose of which produces an antinociceptive effect in a pain model associated with peripheral tissue injury [45]. In the future, it will be important to comprehensively investigate the therapeutic potential of cilnidipine by examining whether orally administered cilnidipine has an analgesic effect at lower doses that have no effect on blood pressure using preclinical models of neuropathic pain. However, considering the fact that the IC50 values of cilnidipine to block L- and N-type Ca^2+^ channels (100 and 200 nM, respectively) [46] are comparable to that to inhibit rP2X7Rs (as demonstrated by our experiments), it could be possible that at a dose of cilnidipine that is effective for neuropathic pain, it may have a lowering effect on blood pressure. Considering this prediction, a possible way to produce a therapeutic effect of cilnidipine on neuropathic pain without lowering blood pressure may be its intrathecal administration, as previously reported in SNX-111, an N-type Ca^2+^ channel blocker [47]. In addition, structural optimization of cilnidipine to increase its selectivity for P2X7R without blocking Ca^2+^ channels may also be a way to develop new analgesics. 

## 5. Conclusions

This is the first study to perform a large-scale and high-throughput screening of clinically approved drugs for P2X7R and identify several compounds, especially cilnidipine, that have potent inhibitory effects on P2X7R, IL-1β release, and mechanical pain hypersensitivity in a model of neuropathic pain. The P2X7R–IL-1β neuroinflammatory pathway has been implicated in chronic pain and various other diseases of the CNS [48]. Recently, the P2X7R antagonist AZ11645373 has been demonstrated to be effective in dampening hyperinflammation and severe influenza disease [49]. Therefore, the newly identified pharmacological effect of cilnidipine could be effective for treating chronic pain and various other inflammatory diseases.

## Data Availability

The data presented in this study are available in insert article or supplementary material here.

## Supplementary Materials

The following are available online at https://www.mdpi.com/2073-4409/10/2/434/s1, Figure S1: A list of 22 compounds that may block or affect Ca^2+^ channels evaluated in chemical screening for rat P2X7R inhibition.
