# Supplementary material for: New Inhibitory Effects of Cilnidipine on Microglial P2X7 Receptors and IL-1β Release: An Involvement in its Alleviating Effect on Neuropathic Pain
Source: Cells. 2021 Feb 18;10(2):434. doi: 10.3390/cells10020434 (PMC7922706; doi:10.3390/cells10020434)
Supplement: Supplementary file 1 [file cells-10-00434-s001.pdf]

cells

MDPI

Compound name	% of control
Cilnidipine	27.6%
Felodipine	>75%
Lomerizine	>85%
Mebeverine hydrochloride	>85%
Nicardipine hydrochloride	>85%
Niguldipine	>85%
Thioridazine hydrochloride	>85%
Amlodipine	>95%
Benidipine HCl	>95%
Calycanthine	>95%
Diltiazem hydrochloride	>95%
Dotarizine	>95%
Flunarizine dihydrochloride	>95%
Lacidipine	>95%
Lercanidipine hydrochloride hemihydrate	>95%
Loperamide hydrochloride	>95%
Manidipine	>95%
Mibefradil dihydrochloride	>95%
Nifedipine	>95%
Nisoldipine	>95%
Perhexiline maleate	>95%
Tolterodine tartrate	>95%

Supplementary Figure 1. A list of 22 compounds that may block or affect Ca^{2+} channels evaluated in chemical screening for rat P2X7R inhibition.
